# Impact of *Clostridium difficile*-associated disease in a regional hospital

**DOI:** 10.1186/1753-6561-5-S6-P184

**Published:** 2011-06-29

**Authors:** P Dolcé, J Blanchette, C Ouellet, K Levesque, H Bernatchez

**Affiliations:** 1 Medical Microbiology and Infectious Diseases, CSSS Rimouski-Neigette, Rimouski, Quebec, Canada; 2 Infection Control, CSSS Rimouski-Neigette, Rimouski, Quebec, Canada

## Introduction / objectives

For the recent years, our institution had an incidence of *Clostridium difficile* associated disease (CDAD) higher than the provincial rate. This study reviewed the burden of CDAD in our hospital.

## Methods

CSSSRN is a tertiary regional 230-beds acute care, with only 34 (15%) single rooms with toilet. Cases from April 2009 to March 2011 were retrieved with Nosokos, a web-base electronic surveillance software used by our Infection Control Team. Data analysis included demographics, risk factors, origin of acquisition, isolation, intra-hospital transfers and complications. The estimated daily costs per case of CDAD were 1000$/inpatient care, 200$/isolation and enhanced environmental disinfection, 300$/transfer.

## Results

A total of 242 episodes of CDAD in patients were observed in 190 patients, including 57 (23,4%) recurrences. Our incidence rate was 9,6/10000 patients-days. Many cases were caused by the hyper-virulent strain NAP1/027. The median age was 75 years (range 2-98) and 57% were women. The origin of acquisition from first episodes were nosocomial 60%, transfer from another facility 9%, non nosocomial 27%, unknown 4%. Hospitalization was required in 181 episodes (75%) for a median of 7 days (range 1-167). Colectomy was performed in 5 patients, death < 30 days occurred in 12% cases . Of 13445 isolation-days, 13,5% were associated with CDAD, for an average of 1,6 CDAD isolations/100 patients-days. Also, 10357 intra-hospital transfers were done and 1,3% were CDAD cases. Using our definitions, the estimated costs of CDAD in our institution were 2 600 000 $ for the 2-year period.

## Conclusion

CDAD caused severe disease, in an elderly population, with a high death rate of 12% and yearly costs of 1 300 000 $ in our institution.

## Disclosure of Interest

P. Dolcé Shareholder of Nosotech, J. Blanchette: None declared, C. Ouellet: None declared, K. Levesque: None declared, H. Bernatchez: None declared.

